# Genipin inhibits rotavirus-induced diarrhea by suppressing viral replication and regulating inflammatory responses

**DOI:** 10.1038/s41598-020-72968-7

**Published:** 2020-09-28

**Authors:** Jong-Hwa Kim, Kiyoung Kim, Wonyong Kim

**Affiliations:** Department of Microbiology, Chung-Ang University College of Medicine, 84, Heukseok-ro, Dongjak-gu, Seoul, 06974 South Korea

**Keywords:** Infectious diseases, Antimicrobials

## Abstract

Rotavirus is the leading cause of acute gastroenteritis among young children worldwide. However, agents specifically designed to treat rotavirus infection have not been developed yet. In this study, the anti-rotavirus and anti-inflammatory effects of genipin, a chemical compound found in the fruit of *Gardenia jasminoides*, were evaluated. Genipin had an antiviral effect against the human rotavirus Wa and SA-11 strains in vitro*,* and it inhibited two distinct stages of the viral replication cycle: attachment and penetration (early stage) in pre-treatment and assembly and release (late stage) in post-treatment. Additionally, genipin downregulated nitric oxide synthase and pro-inflammatory cytokines in lipopolysaccharide-stimulated RAW264.7 cells and rotavirus-infected Caco-2 cells. Oral administration of genipin before and after viral infection with the murine rotavirus epidemic diarrhea of infant mice strain led to a reduced duration of diarrhea and faecal viral shedding and to decreased destruction of the enteric epithelium. Genipin could have potential as a natural compound with preventive and therapeutic effects against infection and colitis caused by rotavirus.

## Introduction

Rotavirus is a genus of viruses in the *Reoviridae* family, which are the leading cause of acute diarrhea among children aged < 5 years^1^. It is estimated that rotavirus gastroenteritis kills nearly 200,000 children each year, mainly in developing countries^2^. Rotavirus infection can cause vomiting, nausea, fever, abdominal cramps, and abdominal watery diarrhea, resulting in symptoms of dehydration^3^. There is no effective specific treatment for rotavirus gastroenteritis. Oral rehydration has been the most commonly employed treatment to mitigate the effects of infection^4^. However, mucosal and systemic immunomodulatory medications, such as active antiviral agents, are often inadequate for treating rotavirus gastroenteritis^5^.

Currently, two licensed rotavirus vaccines, a monovalent human vaccine, Rotarix (GlaxoSmithKline Biologicals, Rixensart, Belgium), and a pentavalent human-bovine reassortant vaccine, RotaTeq (Merck and Co. Inc., West Point, PA, USA), are used in more than 100 countries^6^. The widespread use of these two vaccines has had a significant impact on reducing morbidity, mortality, and hospitalization associated with rotavirus diarrhea globally^7^. However, in terms of the capacity of rotavirus vaccination to prevent severe gastroenteritis, clinical trials have reported efficacy rates of 45–79% in developing countries and less than 87% in developed countries^8–12^. Moreover, unusual rotaviruses (e.g., G9, G11, G12, and P[4] genotypes) have recently been isolated from vaccinated infants, and these strains were generated from human and animal rotavirus reassortment^13–15^.

Therefore, there is a need to find new strategies to control diarrhea and gastroenteritis caused by rotaviruses. There are several natural and synthetic compounds that reduce rotavirus activity. The synthetic compounds 1-3-d-ribofuranosyl-1,2,4-triazole-3-carboxamide (ribavirin), 3-deazaguanine (3-DG), and inosine pranobex have shown inhibitory effects against the simian rotavirus SA11 strain in MA104 cells^16,17^. Natural products from plants, such as black tea (theaflavin compound)^18^, *Stevia rebaudiana* (anionic polysaccharide compound)^19^, Brazilian medicinal plants^20,21^, *Quillaja saponaria*^22^, and dietary plants^23^, have inhibited the absorption or replication of human and animal rotaviruses. However, medicinal plants and natural molecules for preventing and treating rotavirus infections are currently limited.

Genipin, an aglycone derived from geniposide, is a chemical compound found in the natural fruit of *Gardenia jasminoides*. Genipin is also known as a cross-linker for proteins, gelatin, and chitosan, and it has been reported to be an excellent traditional Oriental medicine with various pharmacologic functions, such as anti-inflammatory, anti-apoptotic, anti-microbial, and anti-tumour effects^24–26^. Recent studies have reported that genipin has antiviral activity against human immunodeficiency virus, swine influenza virus H1N1, Epstein–Barr virus, and Kaposi’s sarcoma-associated herpesvirus^24,27–29^. However, the potential antiviral effects of genipin against rotavirus have not been determined. In this study, the anti-rotavirus and anti-inflammatory activities of genipin, as a potential preventive and therapeutic agent against rotavirus infection, were evaluated using human and murine rotavirus strains in in vitro and in vivo models.

## Results

### Cytotoxicity level

The potential cytotoxicity of genipin against macrophage RAW264.7, MA104 cells, and Caco-2 cells, was evaluated by MTT assay as described in the “Materials and methods” section. The cells were incubated with genipin 0–200 µM/mL. As shown in Fig. S1, the viability of the RAW264.7, MA104, and Caco-2 cells was > 90% at genipin concentrations < 180 µM/mL and < 150 µM/mL. The cells maintained in media supplemented with these genipin concentrations continued to divide during the 3 days of inoculation.

### Effects of NO, PGE_2_, and pro-inflammatory cytokine generation

The inhibitory effects of genipin on accumulated production of LPS-induced NO and PGE_2_ were determined in the cell supernatant of macrophage RAW264.7 cells treated with genipin at concentrations of 10, 50, 100, and 150 µM/mL in the presence of 0.1 μg/μL of LPS. Compared with the control of LPS-induced NO, accumulated NO and PGE_2_ production in the cell supernatant treated with genipin was significantly inhibited at 50–150 µM/mL (Fig. S2A,B). In particular, treatment with genipin 50 µM/mL resulted in NO and PGE_2_ production rates of 12% and 30%, respectively, relative to the untreated control. The levels of IL-6, IL-10, and IL-1β were significantly inhibited at genipin concentrations of 50–150 µM/mL. With 150 µM/mL of genipin, the IL-6 level was 4.0 pg/mL (compared with untreated control, 16.3 pg/mL) (Fig. S2C), the IL-10 level was 14.8 pg/mL (compared with untreated control, 84.8 pg/mL) (Fig. S2D), and the IL-1β level was 10.8 pg/mL (compared with untreated control, 43.9 pg/mL) (Fig. S2E). In contrast, the TNF-α level was slightly inhibited. At a genipin concentration of 150 µM/mL, the TNF-α level was 18.6 pg/mL (compared with untreated control, 21.0 pg/mL) (Fig. S2F).

### Inhibitory effects of genipin against inflammation by rotavirus infection

**Figure 1:**
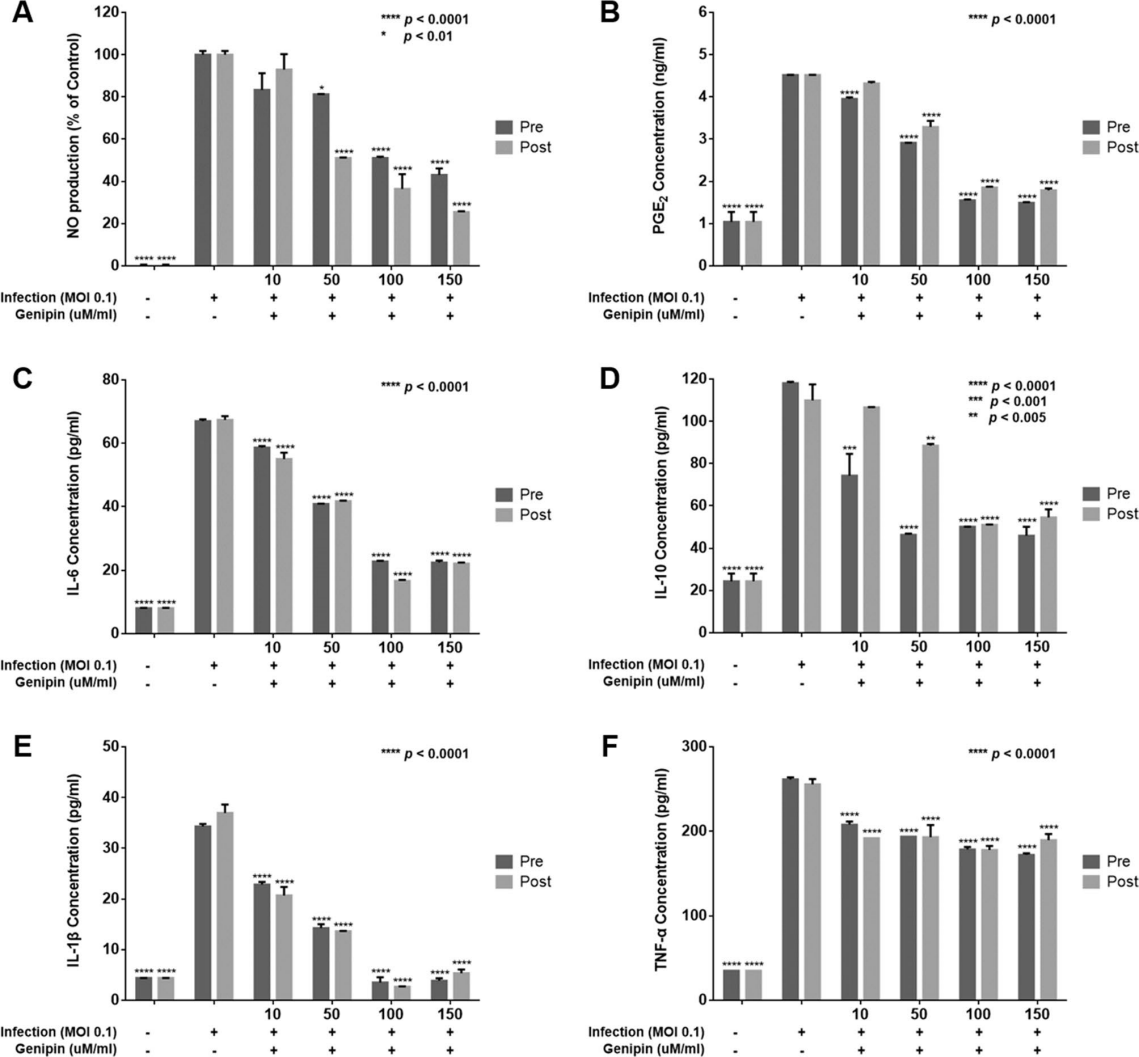
Inhibition effects of genipin on NO, PGE_2_, and pro-inflammatory cytokines in Caco-2 cells. Genipin suppressed rotavirus-infected (**A**) NO and (**B**) PGE_2_ in Caco-2 cells. Effect of genipin on suppression of rotavirus-infected production of pro-inflammatory cytokines, (**C**) IL-6, (**D**) IL-10, (**E**) IL-1β, and (**F**) TNF-α in Caco-2 cells. Caco-2 cells were pre- and post-treated with genipin at different concentrations of 10, 50, 100, and 150 µM/mL and incubated for 24 h. Untreated control cells were inoculated with fresh media only. Data are presented as mean ± SEM; **p < 0.005, ***p < 0.001, ****p < 0.0001.

To evaluate the inhibitory effects of genipin against inflammation by rotavirus infection, NO and PGE_2_ were measured in virus-infected Caco-2 cell supernatant fluid (Fig. 1). The Caco-2 cells infected with rotavirus were used as a positive control. NO and PGE_2_ production were inhibited at genipin concentrations of 50–150 µM/mL in both pre- and post-treatment, compared with the positive control (Fig. 1A,B). The level of pro-inflammatory cytokines was significantly inhibited at genipin concentrations of 50–150 µM/mL in both pre- and post-treatment contexts. In detail, at a genipin concentration of 100 µM/mL, the IL-6 level was 22.7 pg/mL in pre-treatment and 16.6 pg/mL in post-treatment (compared with positive control, 67.3 pg/mL) (Fig. 1C), the IL-10 level was 50.0 pg/mL in pre-treatment and 51.0 pg/mL in post-treatment (compared with positive control, 118.0 pg/mL) (Fig. 1D), the IL-1β level was 3.5 pg/mL in pre-treatment and 2.6 pg/mL in post-treatment (compared with positive control, 36.9 pg/mL) (Fig. 1E), and the TNF-α level was 178.6 pg/mL in pre-treatment and 177.9 pg/mL in post-treatment (compared with positive control, 261.5 pg/mL) (Fig. 1F). At a genipin concentration of 150 µM/mL, the IL-6 level was 22.4 pg/mL in pre-treatment and 22.0 pg/mL in post-treatment (Fig. 1C), the IL-10 level was 45.8 pg/mL in pre-treatment and 54.5 pg/mL in post-treatment (Fig. 1D), the IL-1β level was 3.8 pg/mL in pre-treatment and 5.3 pg/mL in post-treatment (Fig. 1E), and the TNF-α level was 172.1 pg/mL in pre-treatment and 189.5 pg/mL in post-treatment (Fig. 1F).

### Genipin inhibits rotavirus in a dose-dependent manner

**Figure 2:**
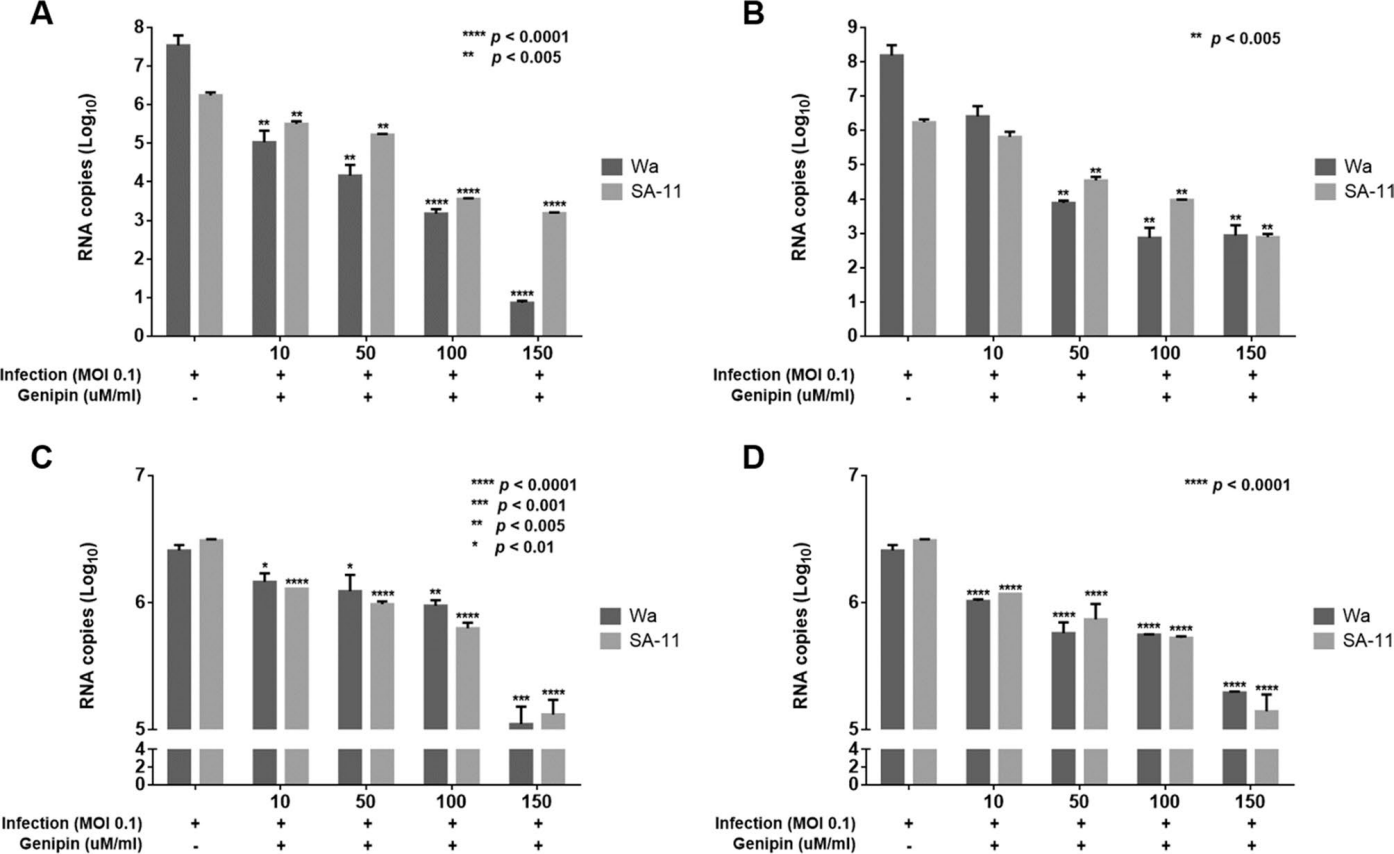
Inhibitory activity of genipin against rotavirus using real-time PCR as a viral titre assay. Genipin at 10, 50, 100, and 150 µM/mL inhibited rotavirus in (**A**) pre-treatment and (**B**) post-treatment on MA104 cells. Caco-2 cells were (**C**) pre- and (**D**) post-treated with genipin at different concentrations. Data are presented as mean ± SEM; *p < 0.01, **p < 0.005, ***p < 0.001, ****p < 0.0001.

The inhibitory effects of genipin against the human rotavirus Wa strain and simian rotavirus SA-11 strain in MA104 cells were determined by a real-time PCR viral titre assay using different strategies. As expected, viral titre was significantly reduced by genipin treatment in a dose-dependent manner. Genipin pre-treatment significantly reduced the Wa strain viral titre (5.02 log of 10 µM, 4.16 log of 50 µM, 3.17 log of 100 µM, and 0.86 log of 150 µM) and SA-11 strain viral titre (5.50 log of 10 µM, 5.22 log of 50 µM, 3.55 log of 100 µM, and 3.18 log of 150 µM), compared with untreated control (7.53 log and 6.23 log, respectively) in MA104 cells (Fig. 2A). Post-treatment also significantly reduced Wa strain viral titre (6.14 log of 10 µM, 3.88 log of 50 µM, 2.86 log of 100 µM, and 2.94 log of 150 µM) and SA-11 strain viral titre (5.8 log of 10 µM, 4.53 log of 50 µM, 3.97 log of 100 µM, and 2.88 log of 150 µM), compared with untreated control (8.18 log and 6.23 log, respectively) in MA104 cells (Fig. 2B).

To assess whether genipin treatment could inhibit rotavirus Wa and SA-11 infectivity in a dose-dependent manner, we used Caco-2 cells for the relevant antiviral assay. Genipin pre-treatment significantly reduced Wa strain viral titre (6.16 log of 10 µM, 6.09 log of 50 µM, 5.97 log of 100 µM, and 5.04 log of 150 µM) and SA-11 strain viral titre (6.11 log of 10 µM, 5.98 log of 50 µM, 5.79 log of 100 µM, and 5.11 log of 150 µM) compared with untreated control (6.41 log and 6.49 log, respectively) in Caco-2 cells (Fig. 2C). The post-treatment assay also significantly reduced Wa strain viral titre (6.01 log of 10 µM, 5.75 log of 50 µM, 5.74 log of 100 µM, and 5.29 log of 150 µM) and SA-11 strain viral titre (6.06 log of 10 µM, 5.86 log of 50 µM, 5.72 log of 100 µM, and 5.14 log of 150 µM), compared with untreated control (6.41 log and 6.49 log, respectively) in Caco-2 cells (Fig. 2D).

### Inhibitory impact of genipin on rotavirus plaque formation in MA104 cells

Genipin was evaluated for anti-rotavirus activity in a plaque assay (Fig. S3). The wells pre-treated with genipin showed significantly lower plaque numbers at concentrations of 10, 50, 100, and 150 µM compared with untreated control wells. Additionally, the wells treated with genipin after viral infection exhibited significantly lower plaque numbers at concentrations of 50, 100, and 150 µM.

### Genipin inhibits rotavirus in an MOI-dependent manner

**Figure 3:**
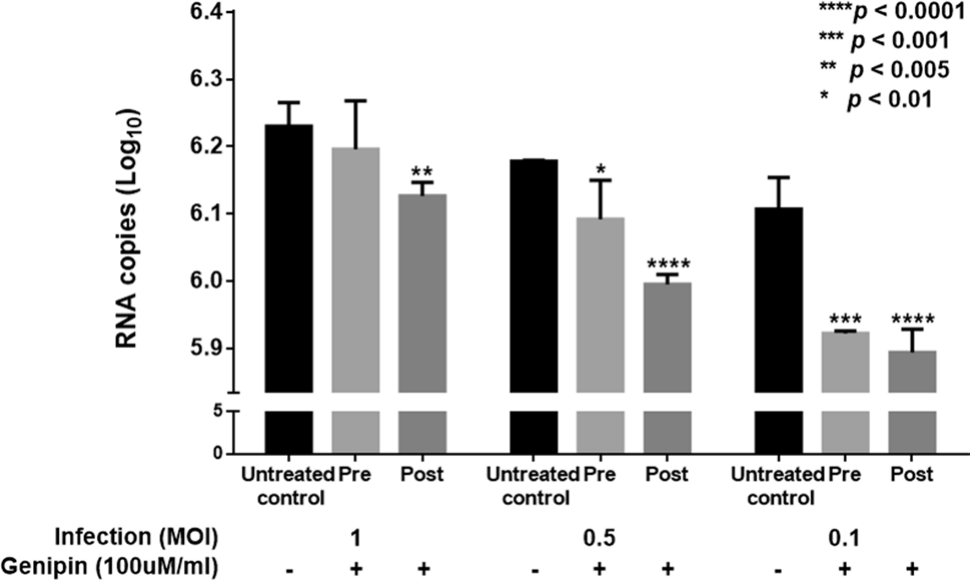
Inhibitory activity of genipin against rotavirus in MOI-dependent manners. Genipin at 100 µM/mL was pre- and post-treated with different MOIs of 0.1, 0.5, and 1. Control was infected rotavirus without genipin. Pre; pre-treatment, Post; post-treatment. Data are presented as mean ± SEM; *p < 0.01, **p < 0.005, ***p < 0.001, ****p < 0.0001.

MA104 cells were infected with multiplicities of infection (MOIs) of 0.1, 0.5, and 1 (Fig. 3). In genipin pre- and post-treatment, infectious virus particle production was significantly inhibited at MOIs of 0.1, 0.5, and 1. Genipin pre-treatment significantly reduced viral titre (5.92 log of MOI 0.1, 6.09 log of MOI 0.5, and 6.19 log of MOI 1). Genipin post-treatment also significantly reduced viral titre (5.89 log of MOI 0.1, 5.99 log of MOI 0.5, and 6.12 log of MOI 1), compared with untreated control (6.10 log of MOI 0.1, 6.17 log of MOI 0.5, and 6.32 log of MOI 1).

### Effects on the viricidal activity of genipin

**Figure 4:**
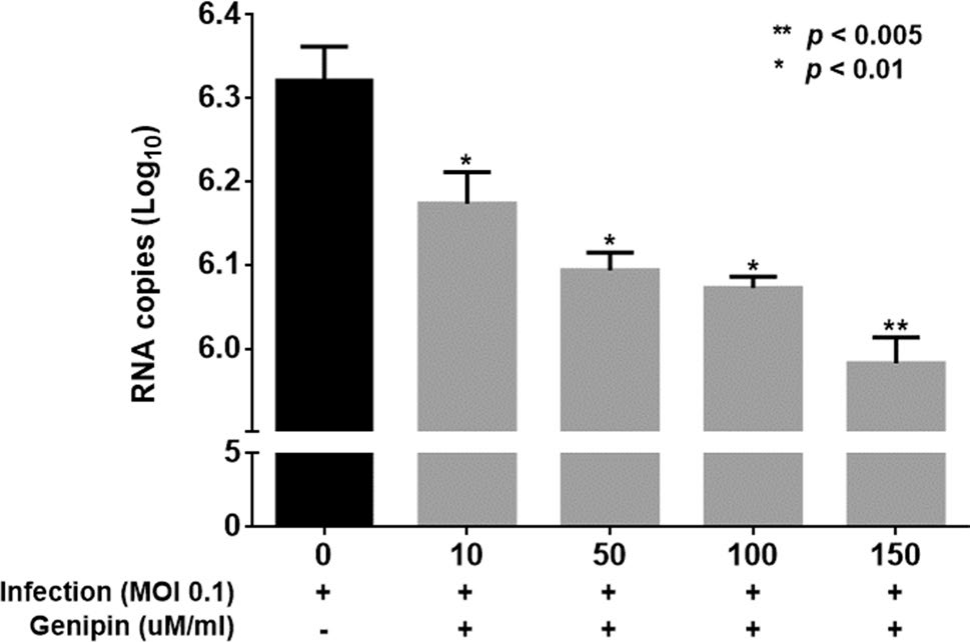
Effects on the viricidal activity of genipin. Viricidal activity of genipin against rotavirus in MA104 cells. Viral infectivity was determined by qPCR. Data are presented as mean ± SEM; *p < 0.01, **p < 0.005.

The viricidal activity of genipin against the human rotavirus Wa strain was determined via a real-time PCR viral titre assay using MA104 cells (Fig. 4). The genipin inhibited the Wa strain viral activity (6.17 log of 10 µM, 6.09 log of 50 µM, 6.07 log of 100 µM, and 5.98 log of 150 µM) relative to untreated control (6.32 log).

### The effect of the genipin kinetics on rotavirus replication

**Figure 5:**
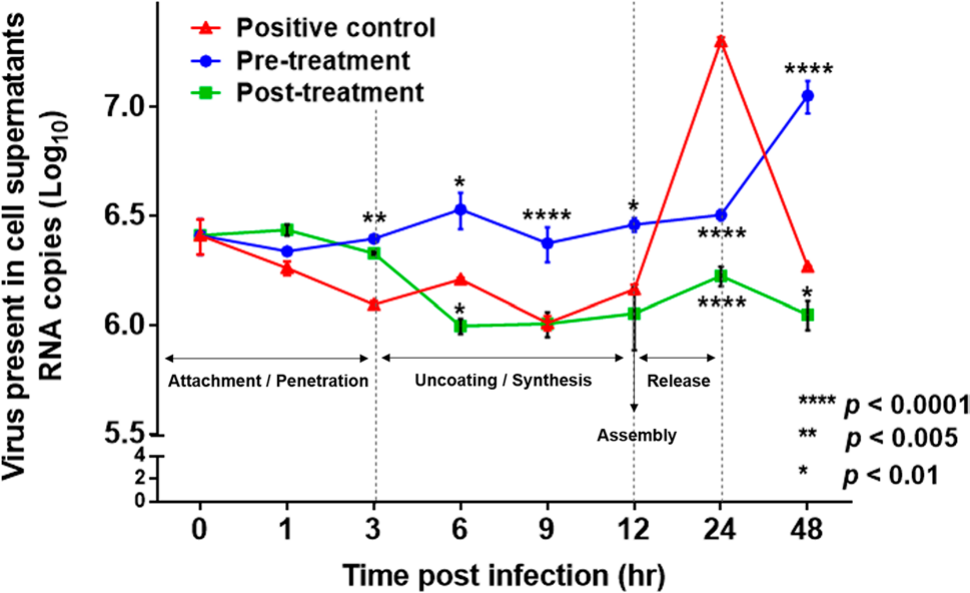
The effect of the genipin kinetics of rotavirus release. Genipin pre- and post-treatment affects the kinetics of rotavirus release. Rotavirus (Wa strain)-infected MA104 cell supernatants were harvested at selected times during the rotavirus replication cycle (0 to 48 h). Data are presented as mean ± SEM; *p < 0.01, **p < 0.005, ****p < 0.0001.

MA104 cells were infected with the Wa strain (MOI 0.1). To confirm viral infection into the MA104 cells, cell supernatant was collected at different times post-infection (pi), ranging from 0 to 48 h. Viral titre was confirmed via real-time PCR. As shown in Fig. 5, genipin pre-treatment (100 µM/mL) was associated with inhibition during attachment and penetration (early stage) until 3 h pi and delayed assembly and release (late stage) at 24 and 48 h pi, respectively, compared with the genipin-untreated control (12 and 24 pi). Genipin post-treatment was associated with inhibition in the late stage; assembly until 12 h pi and viral release at 24 h pi. Additionally, the amount of total viral release was significantly higher in genipin-untreated controls compared with pre- and post-treatment with genipin at 24 h pi.

### Impact of genipin on EDIM-induced diarrhea

**Figure 6:**
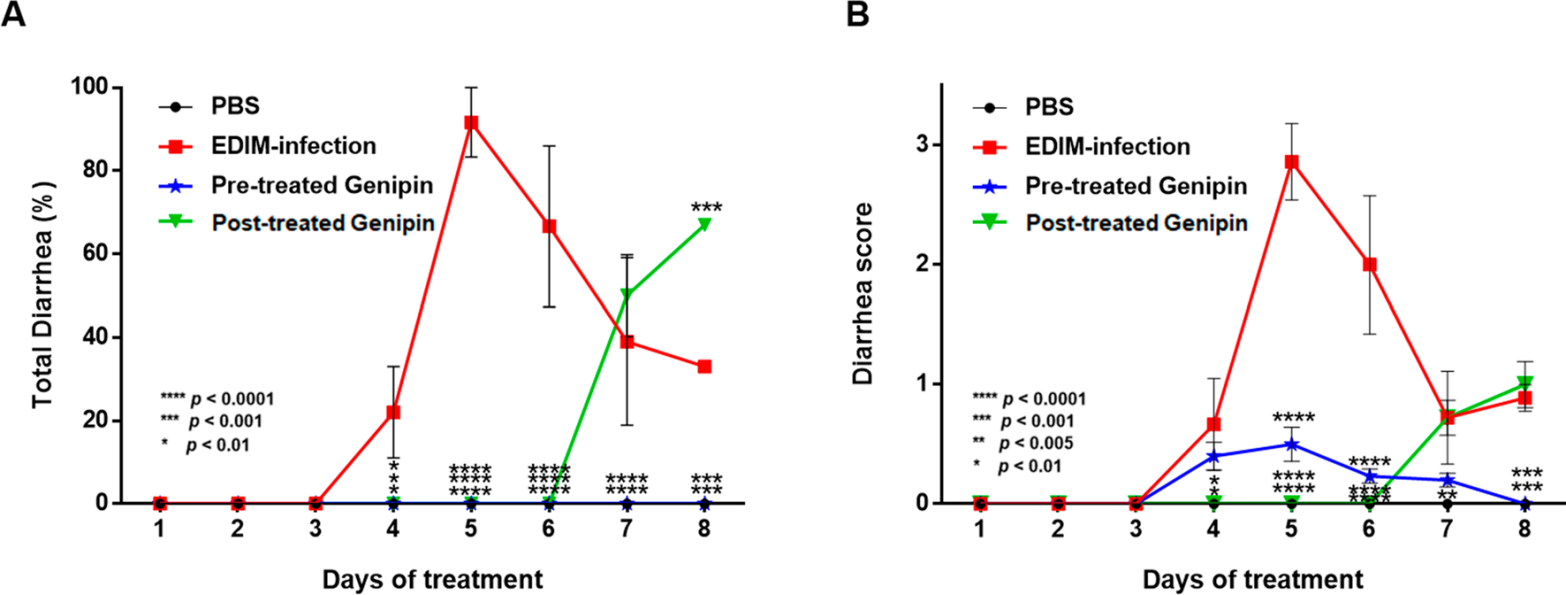
Effects of genipin treatment in vivo. (**A**) Percentage of total diarrhea; (**B**) diarrhea score alterations in EDIM-infected neonatal mice. The percentage of total diarrhea in EDIM-infected neonatal mice was calculated according to the frequency of diarrhea, and the diarrhea score was determined according to the colour and shape of stool samples collected each day. Black, PBS group; blue, EDIM-infected group; red, pre-treated with genipin group; green, post-treated with genipin group. Percentage and score data are expressed as mean ± SEM. *p < 0.01, **p < 0.005, ***p < 0.001, ****p < 0.0001, compared with the EDIM-infected group.

The impact of genipin on EDIM-induced diarrhea in neonatal mice was tested at a genipin concentration of 100 µM/mouse. At 20,000 PFU/mouse, the mice pre-treated with genipin (0%; *p* < 0.05) and those post-treated with genipin (0%; *p* < 0.05) had a significantly lower diarrhea score than the mice in the EDIM group (22.0%) from day 4 after infection (Fig. 6A). On days 7–8, there were significant differences between the mice pre-treated and post-treated with genipin. The incidence of diarrhea among pre-treated mice (0%) was significantly lower than that among post-treated mice (50% on day 7 and 67% on day 8). Specifically, none of the mice pre-treated with genipin had diarrhea, compared with 100% of the mice with acute diarrhea and severe dehydration in the EDIM group after infection. The diarrhea score among mice post-treated with genipin (0; *p* < 0.05) was significantly lower than that among mice in the EDIM group (0.67) on day 4 after infection (Fig. 6B). On days 5–6, the diarrhea scores showed significant differences (*p* < 0.0001) in both the pre-treated (0.50 and 0.23, respectively) and post-treated groups (0 and 0.72, respectively), compared with the EDIM group (2.86 and 2.00, respectively). All groups showed decreased diarrhea scores on day 8. Interestingly, mice post-treated with genipin had a diarrhea score of 0 until day 6, and a score ≤ 1 on days 7–8.

### Viral shedding

**Figure 7:**
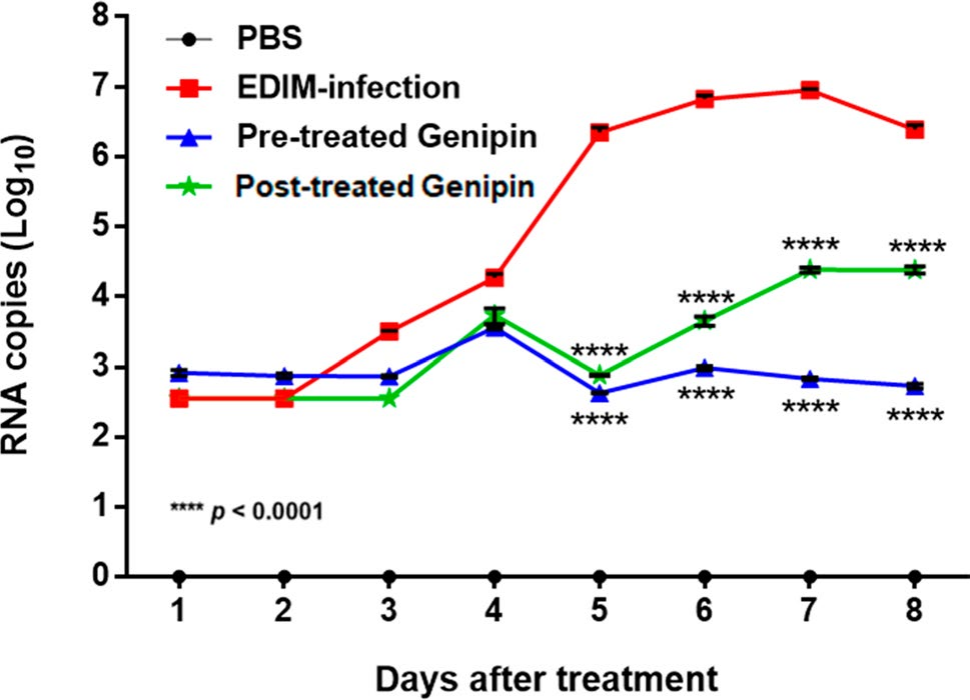
Anti-rotavirus effect of genipin on viral RNA shedding titre. The experiments were evaluated for 8 days, and viral RNA copies were determined by qPCR. Black, PBS group; blue, EDIM-infected group; red, pre-treated with genipin group; green, post-treated with genipin group. Percentage and score data are expressed as mean ± SEM. ****p < 0.0001, compared with EDIM-infected group.

We wanted to confirm whether these approaches also influenced viral load. Total cellular RNA levels isolated from the faeces of neonatal mice pre-treated or post-treated with genipin were analysed by real-time RT-PCR to obtain a number of RNA copies of the EDIM VP6 gene (Fig. 7). From days 1–4, no significant differences in viral RNA shedding titre were observed in the four groups. On day 7, the viral RNA shedding titre in the EDIM group reached its peak by 6.95 log. In contrast, the viral RNA shedding titres in mice pre-treated (2.83 log; *p* < 0.0001) or post-treated with genipin (4.38 log; *p* < 0.0001) were significantly lower than that in the EDIM group. There were statistically significant differences (*p* < 0.005) between the pre-treated and post-treated groups on days 6–8.

## Discussion

Rotavirus is still the leading cause of acute gastroenteritis among young children worldwide. Approximately 95% of children experience at least one rotavirus infection by the age of 5 years^30^. Since antibiotic treatment is nonspecific for rotavirus infection, vaccination is the most important strategy recommended by the World Health Organization to protect children against rotavirus-related hospitalization and death^31^. According to a recent study, only 53 countries offer rotavirus vaccines through their national immunization programs^32^.

The efficacy of rotavirus vaccines is < 90%, so new preventive and therapeutic strategies need to be developed. Our data showed that rotavirus-infected MA104 cells treated with different concentrations of genipin secreted decreased levels of different inflammatory markers (IL-6, IL-10, IL-1β, and TNF-α); we also observed a genipin effect against the inflammatory responses of LPS-induced macrophage RAW264.7 cells. IL-6, IL-1β, and TNF-α are pro-inflammatory cytokines known as triggers of pathologic pain^33,34^.

Previous studies have indicated that some natural products and their secondary metabolites, such as pectic polysaccharides (*Panax ginseng*), epigallocatechin gallate, and theaflavin (green tea) have inhibitory effects against rotavirus^35,36^. However, these studies showed no inhibitory effects against rotavirus infection in either pre- or post-treatment. In the present study, pre- and post-treatment with genipin was associated with inhibitory effects against the human rotavirus Wa and SA-11 strains in MA104 and Caco-2 cells. Plaque numbers and quantification of viral titre via real-time PCR indicated significant decreases in viral infection associated with both pre- and post-treatment with genipin. These results indicate that genipin can be used in the prevention and treatment of rotavirus infection.

Moreover, anti-rotavirus activity was dose-dependently and MOI-dependently affected by genipin in both pre- and post-treatment contexts. Genipin also suppressed rotavirus replication at two distinct stages of the viral replication cycle: the rotavirus RNA replication cycle in MA104 cells was significantly inhibited at the early stage in pre-treatment and the late stage in post-treatment. These results are supported by previous studies, which indicated that kuraridin isolated from *Sophora flavescens* and iridoid glycosides extracted from *Fructus Gardeniae* have inhibited reovirus and influenza virus by replication cycles in pre- and post-treatment^37,38^. Additionally, genipin showed viricidal effects against rotavirus. Anti-rotaviral activity (e.g., inhibiting viral replication and viricidal effects) has previously been reported in association with natural products, including against porcine rotavirus (G5P[7]) and bovine rotavirus (G8P[7])^39,40^.

We also evaluated the inhibitory effects and antiviral activity of genipin against the rotavirus EDIM strain in a murine model. The EDIM group exhibited clinical symptoms of rotavirus infection and high viral shedding, whereas both the pre-treated and post-treated groups exhibited significantly reduced diarrhea incidence and faecal viral shedding. These results are supported by previous in vivo studies, which showed that *Glycyrrhiza uralensis* and *Oryza sativa*, and a combination of *Sophora flavescens* and stevioside, have marked anti-rotavirus effects after induction of rotavirus diarrhea^41–43^. However, these studies investigated only preventive aspects. Taken together, our results demonstrate that genipin may be applied as a potent medication for preventing and curing rotavirus diarrhea in humans and animals.

**Figure 8:**
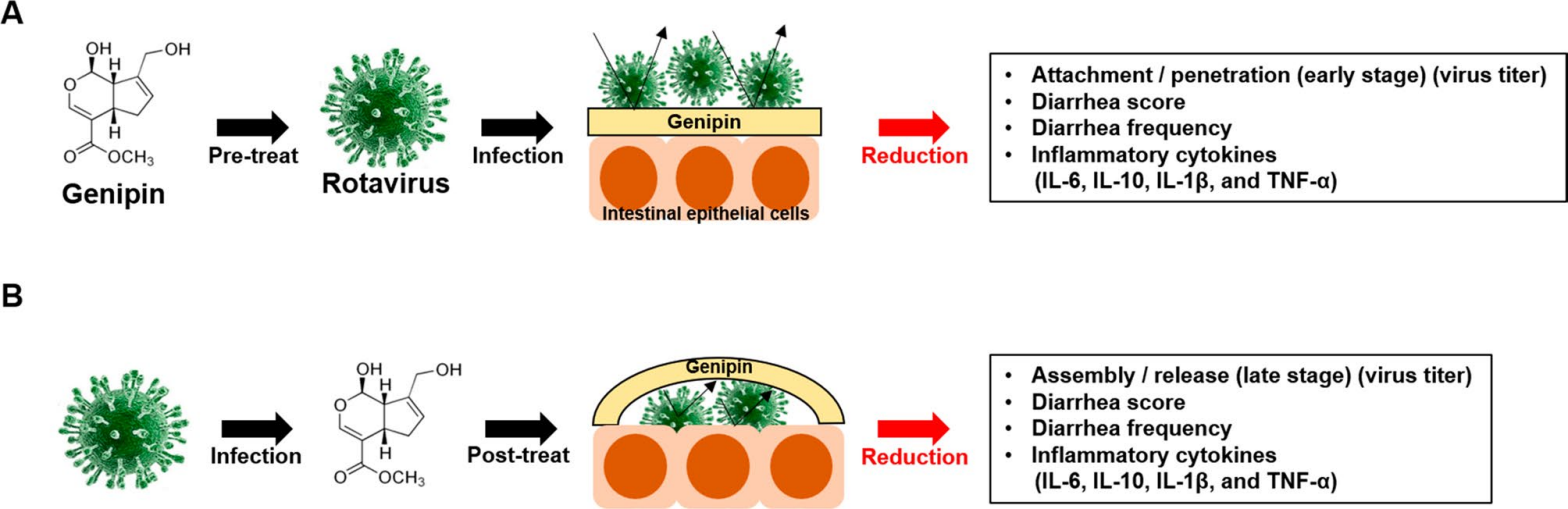
Schematic diagram of genipin experiments. (**A**) Pre-treatment with genipin before rotaviral infection and (**B**) post-treatment with genipin after infection suppressed viral replication cycle in intestinal epithelial cells, respectively, and both decreased diarrhea frequency and score as well as inflammatory cytokine levels.

In conclusion, genipin showed anti-inflammatory and antiviral activities against rotavirus in vitro and in vivo, which led to significantly reduced diarrhea incidence and suppressed viral shedding, by inhibiting the replication cycle (Fig. 8). Therefore, our results suggest that further studies are required for in vitro evaluation of various rotavirus types and for preclinical evaluation of genipin to apply new therapeutic strategies against rotavirus infection.

## Materials and methods

### Reagents

Genipin powder (≥ 98% high-performance liquid chromatography purity, G4796) was purchased and diluted in dimethylsulfoxide (DMSO; Sigma-Aldrich, St. Louis, MO, USA). The mouse interleukins (IL-6, IL-10, and IL-1β), tumor necrosis factor alpha (TNF-α), and prostaglandin E_2_ (PGE_2_) were purchased from R&D Systems (Minneapolis, MN, USA). Cell culture products of alpha minimum essential medium (alpha-MEM), Dulbecco’s modified eagle medium (DMEM), fetal bovine serum (FBS), and gentamicin were purchased from Gibco BRL Life Technologies (Grand Island, NY, USA).

### Cells and viruses

Murine macrophage RAW264.7 cells were obtained from the Korean Cell Line Bank (Seoul, Republic of Korea) for use in the cytotoxicity assay, nitrite measurement, and cytokine analysis. MA104 and Caco-2 cells were used for infection and cultivation of the human rotavirus Wa and SA-11 strains and the murine rotavirus epidemic diarrhea of infant mice (EDIM) strain. RAW264.7 cells were cultured in DMEM, MA104 cells in alpha-MEM containing 5% FBS, and Caco-2 cells in MEM containing 20% FBS at 37 °C in a carbon dioxide (CO_2_) incubator (Thermo Fisher Scientific; Waltham, MA, USA).

### Cytotoxicity assay

The cytotoxicity of genipin against macrophage RAW264.7, MA104, and Caco-2 cells was determined by MTT assay (Mosmann, 1983). Briefly, 100 µL of cell suspension containing 5 × 10^4^, 3 × 10^4^, and 3 × 10^4^ of RAW264.7, MA104, and Caco-2 cells, respectively, was seeded into each well of a 96-well plate and incubated for 24 h. The cells were then treated with genipin at serial concentrations of 10 to 200 µM/mL. After 24 h of incubation, 5 µL of 5 mg/mL MTT reagent (Sigma-Aldrich) was added to each well and incubated at 37 °C for 4 h. The media were then carefully removed, and 150 µL of DMSO was added. The plates were covered with tinfoil and agitated on an orbital shaker for 15 min. The optical density at 590 nm was measured using a NanoQuant spectrophotometer (Infinite 200; Tecan, Männedorf, Switzerland).

### Measurement of nitrite level

To confirm the genipin effects for inflammation, macrophage RAW264.7 cells were cultured in a 24-well plate at a density of 5 × 10^5^ and incubated at 37 °C in the presence of 5% CO_2_ for 24 h. The cells were then treated with 0.1 μg/μL of lipopolysaccharide (LPS) in the presence of genipin at serial concentrations of 10, 50, 100, and 150 µM/mL and incubated at 37 °C for 24 h. Additionally, to evaluate nitrate concentration for infection of the human rotavirus Wa by treatment with genipin, Caco-2 cells were cultured in a 96-well plate at a density of 3 × 10^4^ and incubated at 37 °C in the presence of 5% CO_2_ until forming a confluent monolayer. The procedures to investigate the antiviral activity of genipin against the human rotavirus Wa strain in Caco-2 cells were carried out as follows: (1) pre-treatment—Caco-2 cells were pre-treated with various concentrations of genipin, followed by washes twice with alpha-MEM; the cells were then infected with rotavirus at a multiplicity of infection (MOI) of 0.1 in the presence of 5 µg/mL of trypsin at 37 °C for 1 h; the unbound viruses were then washed twice, and the cells were incubated for 24 h until the cytopathic effect (CPE) appeared; and (2) post-treatment—Caco-2 cells were infected with rotavirus at an MOI of 0.1 in the presence of 5 µg/mL trypsin at 37 °C for 1 h; the unbound viruses were then washed twice with alpha-MEM, and the cells were then treated with different concentrations of genipin until the CPE appeared. After 24 h of incubation, the supernatant was collected by centrifugation at 1000×*g* for 5 min, aliquoted, and stored at − 80 °C. The nitrite was measured using the Griess reagent system (Sigma-Aldrich, St. Louis, MO, USA). An equal volume of culture supernatant was mixed with Griess reagent and incubated at 25 °C for 10 min. The nitrite concentration was determined by measuring absorbance at 540 nm using a microplate reader.

### Measurement of IL-6, IL-10, IL-1β, TNF-α, and PGE_2_ cytokine levels

Macrophage RAW264.7 and Caco-2 cells were cultured in a 24-well plate at a density of 5 × 10^5^ and incubated at 37 °C with 5% CO_2_ for 24 h. The cells were then treated with 0.1 μg/μL of LPS in the presence of genipin at serial concentrations of 10, 50, 100, and 150 µM/mL and incubated for 24 h. The supernatant was collected by centrifugation at 1000×*g* for 5 min, aliquoted, and stored at − 80 °C. To determine the inflammatory inhibition effect of genipin, the Caco-2 cell supernatant was obtained using the same method as above—the nitrate concentration evaluation methods (1) and (2). The IL-6, IL-10, IL-1β, TNF-α, and PGE_2_ in the supernatant were determined by an ELISA kit according to the manufacturer’s instructions (BD Biosciences; San Diego, CA, USA). Briefly, 50 µL of each assay diluent and 50 µL of each sample were added to each well and incubated for 2 h at 25 °C. After washing five times with 400 µL of wash buffer, 100 µL of mouse conjugate was added and incubated for 2 h at 25 °C. Finally, 100 µL of substrate solution was added and incubated for 30 min at 25 °C in the dark, followed by adding 100 µL of stop solution. The optical density at 450 nm was determined using a microplate reader. The cytokine concentration of each sample was determined by comparing to a created standard curve according to the manufacturer’s instructions (BD Biosciences).

### Anti-rotavirus activity in vitro in different concentrations of genipin

The antiviral activity of genipin was evaluated against the human rotavirus Wa strain in MA104 cells as follows: (1) pre-treatment—MA104 monolayer was pre-treated with different concentrations of genipin (10, 50, 100, and 150 µM/mL for 24 h), followed by two washes with alpha-MEM; the cells were then infected with rotavirus at an MOI of 0.1 in the presence of 5 µg/mL trypsin at 37 °C for 1 h; the unbound viruses were then washed twice, and the cells were incubated for 24 h until the CPE appeared; and (2) post-treatment—MA104 cells were infected with rotavirus at an MOI of 0.1 in the presence of 5 µg/mL trypsin at 37 °C for 1 h; the unbound viruses were then washed twice with alpha-MEM, and the cells were then treated with different concentrations of genipin (10, 50, 100, and 150 µM/mL) for 24 h, or until the CPE appeared. After the experiments, cells were harvested by freezing and thawing three times and centrifuged at 3000×*g* for 5 min, and the supernatant was stored at − 80 °C for quantitative polymerase chain reaction (qPCR) analysis. To validate the possibility of genipin anti-rotavirus activity, the simian strain SA-11 together with the Wa strain were evaluated in MA104 and Caco-2 cells. These experiments were performed using the same methods as above—the genipin antiviral activity methods (1) and (2). MA104 and Caco-2 cells untreated with genipin were used as a control.

### Anti-rotavirus activity in vitro in an MOI-dependent manner

To evaluate the efficacy of genipin against rotavirus infection, rotavirus stock was serially diluted in MOIs of 0.1, 0.5, and 1. MA104 cells were infected with different rotavirus MOIs. The minimal genipin concentration for anti-rotavirus activity was determined to be 100 µM/mL. These experiments were performed using the same methods as above—the genipin antiviral activity methods (1) and (2).

### Genipin kinetics of rotavirus cell release

The rotavirus replication cycle was determined as previously described, with a few modifications^44–47^. Confluent monolayers of MA104 cells grown in a 96-well plate were washed with alpha-MEM and infected with the human rotavirus Wa strain at an MOI of 0.1. The virus was adsorbed to cells for 1 h at 37 °C. After the adsorption period, the inoculum was removed, and alpha-MEM was added. These experiments were performed using the same genipin antiviral activity methods as above, (1) and (2). MA104 cells untreated with genipin were used as a control. The infected cell supernatant was collected at different times (0, 1, 3, 6, 9, 12, 24, and 48 h).

### Rotavirus quantification using qPCR

Viral RNA was extracted using a QIAamp Viral RNA Mini Kit (Qiagen; Valencia, CA, USA), according to the manufacturer’s instructions, and stored at − 80 °C for real-time reverse transcriptase (RT)-PCR analysis. The *VP6* genes of the human rotavirus Wa, SA-11, and murine rotavirus EDIM strains were used as templates for the RT-PCR assay^48^.

### Plaque assay

The quantification of rotavirus to determine the anti-rotavirus activity of genipin was performed using a plaque assay. For the prevention effect, MA104 cells (3 × 10^5^ cells/well) were seeded on a 6-well plate for 3 days and pre-treated with different concentrations of genipin (10, 50, 100, and 150 µM) at room temperature for 1 h. After being washed twice with phosphate-buffered saline (PBS), MA104 cells were infected with 400 plaque-forming units (PFU)/mL of rotavirus (Wa strain) at room temperature for 1 h. For the treatment effect, MA104 cells (3 × 10^5^ cells/well) were seeded on a 6-well plate (Corning; Lowell, MA, USA) for 3 days and infected with 400 PFU/mL of rotavirus at room temperature for 1 h. After being washed twice with PBS, the cells were treated with different concentrations of genipin (10, 50, 100, and 150 µM) at room temperature for 1 h. For the plaque assay, MA104 cells were overlaid with 1.2% agarose (Sigma-Aldrich) containing modified eagle medium (MEM) 2 × (Gibco) supplemented with 5 µg/mL trypsin after being washed twice with PBS. The rotavirus-infected agar-overlaid cells were incubated until plaques formed. The cells were fixed using 4% paraformaldehyde in PBS and 70% ethanol at room temperature for 20 min and stained with crystal violet. The MA104 cells not treated with genipin were used as a control.

### Potential viricidal activity of the genipin in vitro

To perform the in vitro viricidal activity assays to determine the inhibition effects of genipin, MA104 cells were seeded at a density of 3 × 10^4^ onto 96-well plates and incubated at 37 °C with 5% CO_2_ for 24 h. The mixtures, with a tissue culture infective dose 50 (TCID_50_) of rotavirus (Wa strain), were mixed with genipin 10–150 µM/mL and activated for 1 h, then added to each well and incubated at 37 °C for 1 h. The inoculum was removed, the cells were washed with PBS, and alpha-MEM containing 5 µg/mL trypsin was added to each well (200 µL/well). The cells were incubated at 37 °C with 5% CO_2_ for 72 h. Activated rotavirus without treatment was included as controls. Experiments were performed in triplicates.

### Anti-rotavirus activity in vivo

Rotavirus EDIM virus was titrated as 4 × 10^7^ TCID_50_/mL in MA104 cells and 2 × 10^4^ infective dose (ID)_50_/mL in neonatal mice using diarrhea as the endpoint, in accordance with the Reed-Muench formula^49^. Animal studies were conducted according to Korean Food and Drug Administration guidelines. All experimental protocols were approved by the Chung-Ang University Institutional Animal Care and Use Committee of the Laboratory Animal Research Center (IACUC No. 2017-00044). Pregnant BALB/c mice were purchased from Samtako (Osan, Republic of Korea). Neonatal mice (4 days old) were divided into two experiments: (1) pre-treatment; and (2) post-treatment. All experiments included three groups: negative (PBS); positive (EDIM-infection); and experimental (genipin) groups (n = 7 mice per group). The EDIM-infected group was orally infected with the rotavirus EDIM strain for 5 consecutive days with 10 µL of 2 × 10^4^ fluorescent focus units (FFU) of challenge-dose virus.

In the genipin-treated group for pre-treatment, mice were orally pre-treated with genipin 100 µM/mouse for 5 days before infection with the rotavirus EDIM strain and then with a dose of the EDIM virus mixed with 100 µM/mouse of genipin (EDIM plus genipin) for 5 consecutive days. The mixtures were incubated at room temperature for 1 h in sterile screw-capped vials. For post-treatment, neonatal mice were orally inoculated with a dose of the EDIM virus mixed with 100 µM/mouse of genipin (EDIM plus genipin) for 5 consecutive days. An untreated virus-infected control set was prepared by diluting 10 µL (2 × 10^4^ FFU) of virus suspension with 10 µL of PBS (EDIM-infected group), and negative control mice received 20 µL of PBS instead (PBS group). Stool consistency was evaluated on a 5-point scale: severe illness (yellow = 4, brown-yellow = 3, brown = 2, black = 1) and consistency (very liquid = 4, liquid = 3, soft = 2, solid = 1)^49–51^.

The different categories of diarrhea reflected the amount of water lost during rotavirus infection. Diarrhea severity was reported as a diarrhea score, where level 3 was less severe, and level 4 was most severe. Faeces were collected daily, and EDIM viral shedding was assessed by qPCR analysis.

### Statistical analysis

Significant differences among the three groups were calculated using two-way analysis of variance with Dunnett’s post-test for comparison using GraphPad Prism (v.6.0.1) (GraphPad Software Inc., San Diego, CA, USA). Data are expressed as mean ± standard error of the mean (SEM), and *p* values < 0.05 were considered significant.

### Ethical approval

Animal studies were performed according to Korean Food and Drug Administration guidelines, and samples were collected from neonatal mice in accordance with the animal ethical guidelines of Chung-Ang University Institutional Animal Care and Use Committee of the Laboratory Animal Research Center (IACUC No. 2017-00044).

## Supplementary information

Supplementary Information.
