# Supplementary material for: Genipin inhibits rotavirus-induced diarrhea by suppressing viral replication and regulating inflammatory responses
Source: Sci Rep. 2020 Sep 28;10:15836. doi: 10.1038/s41598-020-72968-7 (PMC7522720; doi:10.1038/s41598-020-72968-7)
Supplement: Supplementary file 1 — Supplementary Information. [file 41598_2020_72968_MOESM1_ESM.docx]

**Supplementary information for manuscript:**

**Genipin inhibits rotavirus-induced diarrhea by suppressing viral replication and regulating inflammatory responses**

Jong-Hwa Kim, Kiyoung Kim, and Wonyong Kim^*^

**
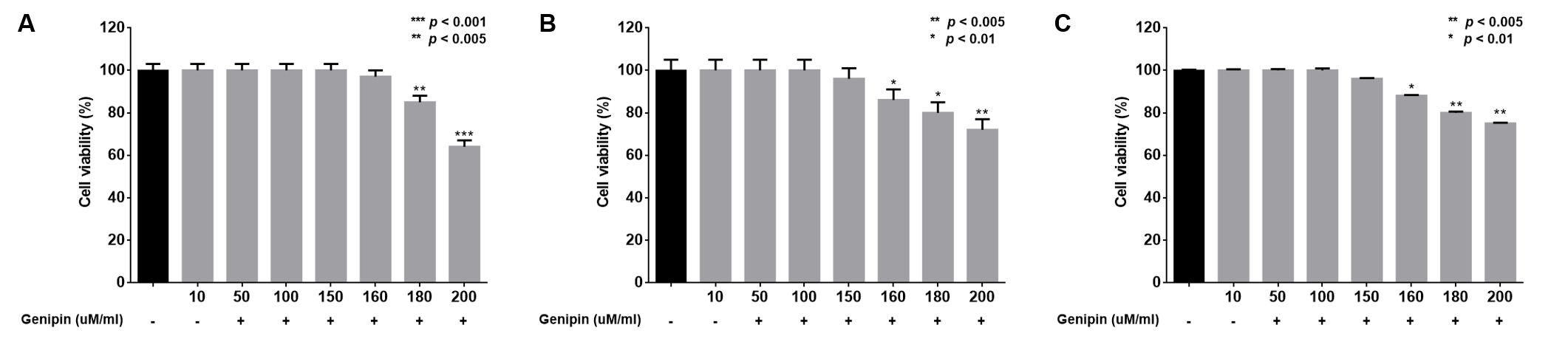
**

**Fig. S1.** **Cytotoxicity effect of genipin on treatment.** (A) RAW264.7, (B) MA104, (C) Caco-2 monolayer cells in 96-well plates were treated with genipin at serial concentrations (0, 10, 50, 100, 150, 160, 180, and 200 µM/mL). Cell viability was determined by MTT assay. Control cells were inoculated with media only. Data are presented as mean ± SEM; **p* < 0.01, ***p* < 0.005, ****p* < 0.001.


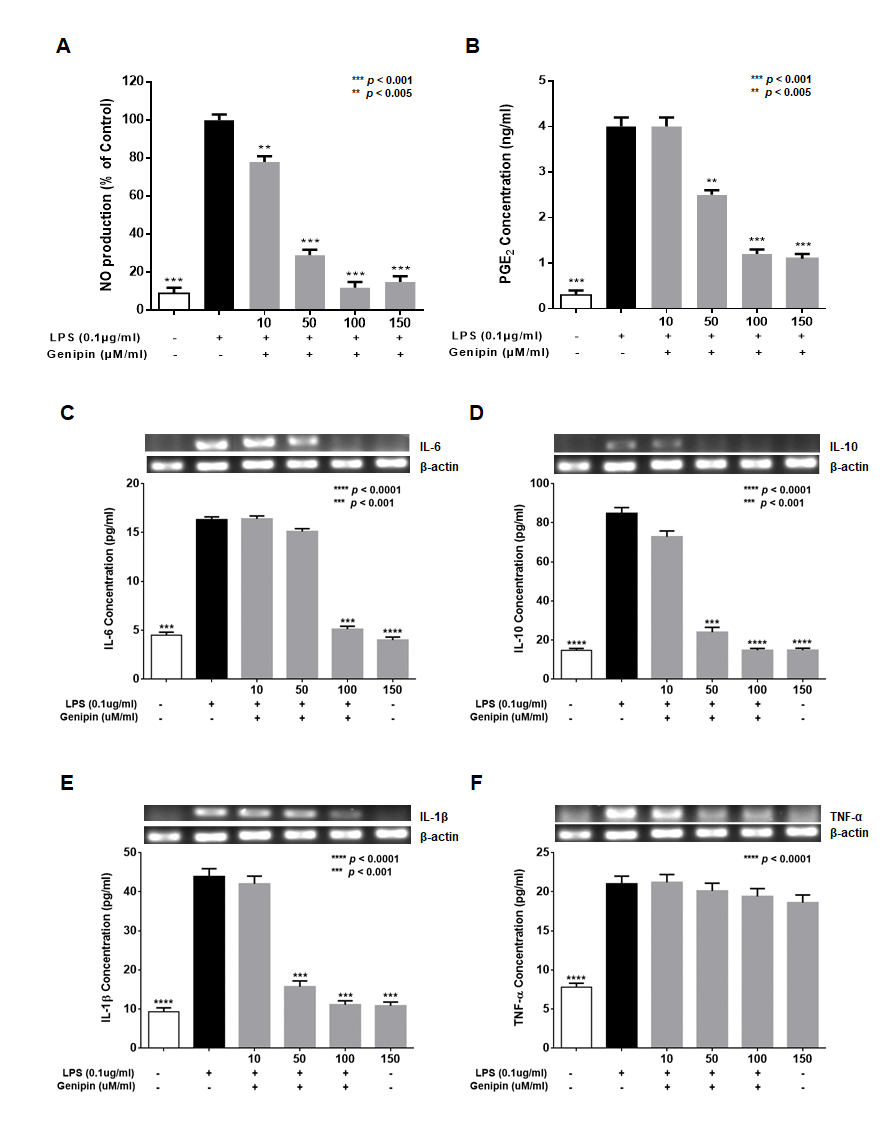


**Fig. S2.** **Suppression effects of genipin on NO, PGE2, and pro-inflammatory cytokines in RAW 264.7 cells.** Genipin suppressed LPS-induced (A) NO and (B) PGE_2_ in RAW 264.7 cells. Cells were treated with 0.1 μg/μL of LPS in the presence or absence of genipin, and the supernatant was collected to evaluate accumulated NO and PGE_2_. Effect of genipin on suppression of LPS-induced production of proinflammatory cytokines, (C) IL-6, (D) IL-10, (E) IL-1β, and (F) TNF-α in RAW264.7 macrophage cells. RAW264.7 cells were pre-treated with 0.1 μg/μL of LPS in the presence of genipin at different concentrations (10, 50, 100, and 150 µM/mL) and incubated for 24 h. Untreated control cells were inoculated with fresh media only. Data are presented as mean ± SEM; ***p*<0.001, ****p*<0.005, *****p*<0.0001.


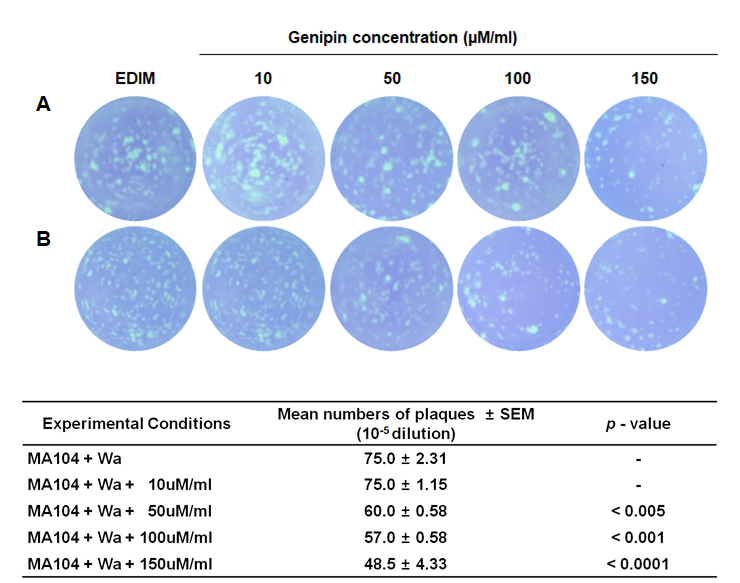


Fig. S3. Inhibitory activity of genipin against rotavirus by plaque assay. MA104 cells were treated with genipin (A) before viral infection and (B) after viral infection at 10, 50, 100, and 150 µM/mL. The human rotavirus Wa-infected group and untreated cells were used as positive controls.
